# Comparing the B and T cell-mediated immune responses in patients with type 2 diabetes receiving mRNA or inactivated COVID-19 vaccines

**DOI:** 10.3389/fimmu.2022.1018393

**Published:** 2022-10-11

**Authors:** Chi-Ho Lee, Victor Gray, Jia Ming Nickolas Teo, Anthony Raymond Tam, Carol Ho-Yi Fong, David Tak-Wai Lui, Polly Pang, Kwok Hung Chan, Ivan Fan-Ngai Hung, Kathryn Choon-Beng Tan, Guang Sheng Ling

**Affiliations:** ^1^ Department of Medicine, School of Clinical Medicine, Li Ka Shing Faculty of Medicine, Queen Mary Hospital, The University of Hong Kong, Hong Kong, Hong Kong SAR, China; ^2^ School of Biomedical Sciences, Li Ka Shing Faculty of Medicine, The University of Hong Kong, Hong Kong, Hong Kong SAR, China; ^3^ Department of Microbiology, School of Clinical Medicine, Li Ka Shing Faculty of Medicine, Queen Mary Hospital, The University of Hong Kong, Hong Kong, Hong Kong SAR, China

**Keywords:** type 2 diabetes, COVID-19 vaccine, CoronaVac, BNT162b2, immunological memory response

## Abstract

Acquiring protective immunity through vaccination is essential, especially for patients with type 2 diabetes who are vulnerable for adverse clinical outcomes during coronavirus disease 2019 (COVID-19) infection. Type 2 diabetes (T2D) is associated with immune dysfunction. Here, we evaluated the impact of T2D on the immunological responses induced by mRNA (BNT162b2) and inactivated (CoronaVac) vaccines, the two most commonly used COVID-19 vaccines. The study consisted of two parts. In Part 1, the sera titres of IgG antibodies against severe acute respiratory syndrome coronavirus 2 (SARS-CoV2) alpha receptor binding domain **(**RBD), their neutralizing capacity, and antigen-specific CD4^+^T and CD8^+^T cell responses at 3-6 months after vaccination were compared between BNT162b2 (n=60) and CoronaVac (n=50) vaccinees with or without T2D. Part 2 was a time-course study investigating the initial B and T cell responses induced by BNT162b2 among vaccinees (n=16) with or without T2D. Our data showed that T2D impaired both cellular and humoral immune responses induced by CoronaVac. For BNT162b2, T2D patients displayed a reduction in CD4^+^T-helper 1 (Th1) differentiation following their first dose. However, this initial defect was rectified by the second dose of BNT162b2, resulting in comparable levels of memory CD4^+^ and CD8^+^T cells, anti-RBD IgG, and neutralizing antibodies with healthy individuals at 3-6 months after vaccination. Hence, T2D influences the effectiveness of COVID-19 vaccines depending on their platform. Our findings provide a potential mechanism for the susceptibility of developing adverse outcomes observed in COVID-19 patients with T2D and received either CoronaVac or just one dose of BNT162b2.

## Introduction

Vaccines are seen as the most effective approach to overcome the ongoing coronavirus disease (COVID-19) pandemic, where 400 million people have been infected,with 6 million fatalities (1). Understanding COVID-19 vaccine efficacy is of paramount importance, especially their ability to protect high risk individuals, such as those with type 2 diabetes who are particularly prone to develop severe complications from severe acute respiratory syndrome coronavirus 2 (SARS-CoV2) infection. Although the reasons by which type 2 diabetes influences infection course are multifactorial, defects in both innate and adaptive immune systems are among the major contributing factors (2–10). Impaired vaccination responses to influenza and hepatitis B vaccines have already been extensively described in patients with type 2 diabetes (11–13). However, the effectiveness of the currently available COVID-19 vaccines among patients with type 2 diabetes remains poorly defined. Given the high global prevalence of type 2 diabetes (14), a thorough analysis of the vaccine responses among individuals with type 2 diabetes is highly warranted.

Vaccine response consists of B- and T-cell mediated humoral and cellular immunity, which produces neutralizing antibodies and eliminates infected cells. Whilst neutralizing antibodies prevent viral entry, cellular response is the key to prevent severe infection. Moreover, since T cell epitopes are less susceptible to antigenic drift, they are more likely to confer longer-term protection against different SARS-CoV2 variants (15, 16). Previous studies that compared COVID-19 mRNA vaccine-induced humoral responses between patients with type 2 diabetes and healthy controls (HCs) have reported inconsistent findings (17–21). Moreover, few studies have evaluated vaccine-induced cellular immunity in patients with type 2 diabetes. Van Praet et al. showed that peripheral blood mononuclear cells (PBMCs) from patients with diabetes produced less interferon gamma (IFNγ) upon SARS-CoV2 glycoprotein stimulation (22). Marfella et al. showed that CD4^+^T cell responses were impaired in patients with type 2 diabetes who had poor glycaemic control (20, 23). Notably, T cell biology is complex with distinct effector and memory T cell subsets performing discrete roles in immunity (24). Furthermore, the vaccine-elicited T cell immunity in patients with type 2 diabetes, in particular their CD8^+^T cells responses, remains undefined. Therefore, we conducted this study to assess the effectiveness of the two most used COVID-19 vaccines: mRNA (BNT162b2) and inactivated (CoronaVac) vaccines among patients with type 2 diabetes (25), focusing on the analysis of their T cell responses, including the impact of type 2 diabetes on T effector and memory subsets differentiation.

## Materials and methods

### Study design

This study consisted of two parts using independent cohorts of COVID-19 vaccinees (**Supplementary Figure 1**).

The first part evaluated the impact of type 2 diabetes on vaccine-elicited immunological memory among BNT162b2 and CoronaVac vaccinees at 3-6 months following their second dose of inoculation. Serum levels of IgG antibodies against SARS-CoV2 alpha-receptor binding domain **(**RBD) and neutralizing antibodies were compared between patients with type 2 diabetes and HCs. In addition, their antigen-specific CD4^+^T and CD8^+^T cell responses were evaluated by stimulating their PBMCs with peptide pools encompassing the N-terminus (S1) and C-terminus (S2) of the SARS-CoV2 spike (S) protein.

The second part was a time-course study investigating the effects of type 2 diabetes on the initial B and T cell responses induced by BNT162b2. Blood samples were collected from an independent cohort of participants at three distinct timepoints: (i) pre-vaccination, (ii) 20 days after the first dose, and (iii) 14-20 days after the second dose of vaccination. Anti-RBD IgG antibody titres were measured. Pre-vaccinated PBMCs were stimulated along with their paired post-vaccination samples for T cell analysis. S-specific T cells were distinguished by their expression of activation-induced markers, followed by an in-depth phenotypic analysis to determine the effects of type 2 diabetes on T cell differentiation.

### Study participants

In this study, all patients with type 2 diabetes were recruited from the Diabetes Clinic of Queen Mary Hospital, Hong Kong, whereas HCs were invited from the community. Inclusion criteria included an age range between 21 and 75 years inclusive. Exclusion criteria consisted of history of malignancy within 5 years, presence of autoimmune disease, concomitant use of immunosuppressants including corticosteroids, recent hospitalization within 3 months or with ongoing infection at the time of blood collection. Moreover, individuals who were pregnant, or reported prior history of SARS-CoV2 infection were also excluded. The study protocol was approved by the Institutional Review Board (IRB) of the University of Hong Kong/Hospital Authority Hong Kong West Cluster (IRB Ref: UW 21-460). All participants provided informed consent prior to any study-related procedures.

### PBMC collection and processing

In all participants, peripheral venous blood samples were collected with their serum stored at -80°C for further assays. PBMC were isolated with Lymphoprep (STEMCELL Technologies), cryopreserved in 90%FBS/10%DMSO and stored in liquid nitrogen for further analysis.

### Measurements of circulating anti-RBD IgG and neutralizing antibodies

Serum levels of IgG antibodies against Wuhan-Hu-1 RBD were measured using the SARS-CoV2 S1 RBD IgG ELISA kit (ImmunoDiagnostics) and acquired on Thermo Varioskan Flash (Thermofisher). Circulating neutralizing antibodies elicited by vaccinations were measured using the SARS-CoV2 sVNT kit (GenScript).

### Intracellular cytokine staining assay

PBMCs were thawed and resuspended in complete media [RPMI supplemented with 10% FBS, 2mM Sodium pyruvate, 2mM L-Glutamine, 10mM HEPES buffer solution, 1% 100X MEM Non-Essential Amino Acids, 1% penicillin-streptomycin and 0.05mM β-mercaptoethanol (Gibco)] at a density of 5x10^6^ cells/ml. Subsequently, 100 μl of cells were plated into a 96-well U-bottom plate and stimulated for 6 days with soluble α-CD28 (1μg/ml) (Biolegend) and PepMix™ SARS-CoV2 S1 or S2 peptide pools at a final concentration of 1μg/ml (JPT; Catalogue no PM-WCPV-S-1). S1 peptide pools covered N-terminal amino acid residues 1–643 of ancestral strain of spike glycoprotein, and contained 158 15-mers that were overlapped by 11 amino acids. S2 peptide pools covered C-terminal amino acid residues 633–1273 of ancestral strain of spike glycoprotein, and contained 156 15-mers that were overlapped by 11 amino acids and one 17-mer at the C terminus. In other words, 157 peptides in total. Cross-reactive T cell epitopes between SARS-CoV2 and common cold HCoVs were present among the S2 peptide pools (26). All samples were supplemented with 20U/ml of recombinant human interleukin-2 (IL-2) (PerproTech) with the media replenished on day 3. Matched unstimulated samples were cultured with α-CD28 and IL-2 alone. On day 6, samples were restimulated with S1 or S2 peptide pools (1μg/ml) for twelve hours. Two hours post-stimulation, antibody targeting CD107a was added to culture along with Monensin A (5μg/ml) and Brefeldin A (2μM) (Biolegend). Surface staining was performed, followed by cell fixation and permeabilization using BD Cytofix/Cytoperm™ (BD Bioscience). Intracellular staining was then performed in 1x Perm/Wash Buffer with antibodies targeting tumour necrosis factor alpha (TNFα), IFNγ and Granzyme B (GZMB).

### S-reactive T cell detection assay

After stimulating the PBMCs with peptide pools as described above, on day 6, samples were restimulated with S1 or S2 peptide pools (1μg/ml) for twelve hours along with Monensin A and Brefeldin A. Cells were stained for 10 minutes at room temperature with Zombie Aqua Fixable Viability Kit (Biolegend) and Human TruStain FcX Fc receptor blocking solution (Biolegend), and washed once in PBS. Surface staining was then performed with antibodies directed against CD3, CD4, CD8, 4-1BB, CD40L, CD200, CCR6, CXCR3, CXCR5, PD-1, CCR7, CD45RA, CD95, CD27 for 30 minutes at 4°C. Cells were then fixed and permeabilised using BD Cytofix/Cytoperm™ (BD Bioscience) prior to staining for intracellular IFNγ for 30 minutes at 4°C. S-reactive T cells were identified by their expression of activation-induced markers (CD40L, CD200, 4-1BB and intracellular IFNγ). S-reactive CD4^+^T cells as CD40L^+^CD200^+^; S-reactive CD8^+^T cells as IFNγ^+^4-1BB^+^; T-follicular-helper (Tfh) as CXCR5^+^; T-helper (Th)1 as CXCR5^-^CXCR3^+^CCR6^-^; Th17 as CXCR5^-^CXCR3^-^CCR6^+^; Th1/17 as CXCR5^-^CXCR3^+^CCR6^+^; central memory (CM) as CD45RA^-^CCR7^+^; effector memory (EM) as CD45RA^-^CCR6^-^ and T stem cell memory (TSCM) as CD45RA^+^CCR7^+^CD27^+^CD95^+^ (27, 28).

### Flow cytometry

Flow cytometry was performed using the LSRFortessa flow cytometer (BD Bioscience) and the data was analysed with Flowjo V10 software (Tree Star Inc). All data from cytokine expression and S-reactive T cell assays were background subtracted using their paired DMSO (vehicle)-stimulated control samples. The limit of detection for S-reactive CD4^+^ T cell responses (0.2%) and S-reactive CD8^+^ T cell responses (0.06%) were calculated using the median 2-fold SD of all negative controls as recently described (29). List of antibodies and dilutions used are shown in **Supplementary Table 1**. Gating strategies are shown in **Supplementary Figure 2**.

### Statistical analysis

All statistical analyses were conducted using GraphPad Prism software 8.01 (GraphPad Software) and IBM SPSS Statistics 26.0 (http://www.IBM.com/SPSS). Kolmogorov-Smirnov and Shapiro Wilk tests were used to test for normality of data. Categorical variables were compared using Chi-square test or Fisher’s exact test, as appropriate, whereas continuous variables were compared using Mann-Whitney U test. Spearman correlation analysis was performed to analyse the correlations between variables. Quantile regression and multivariable logistic regression analyses were performed to evaluate the independent associations of type 2 diabetes with parameters of vaccine-induced immune responses. Variables that were statistically significant in univariate analyses were included in multivariable regression models. In all analyses, statistical significance was defined as a two-sided p-value<0.05.

## Results

### Neutralizing antibodies elicited by CoronaVac, but not BNT162b2, were less maintained in patients with type 2 diabetes

**Figure 1:**
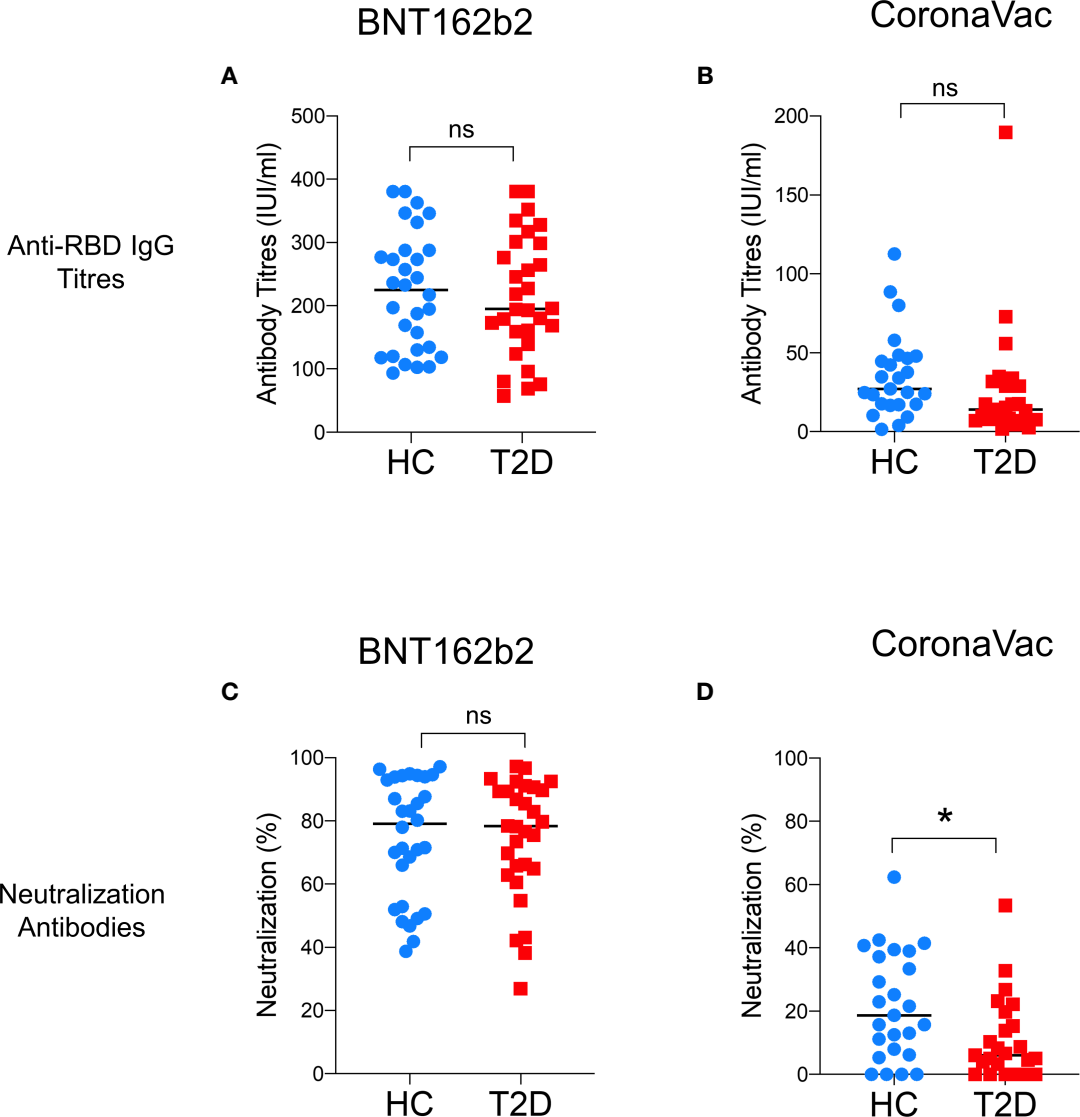
Levels of anti-RBD IgG and neutralizing antibodies 3-6 months following BNT162b2 and CoronaVac vaccination. Serum titres of anti-RBD IgG and neutralizing antibodies measured at 3-6 months following the second dose of BNT162b2 (n=30/group) **(A, C)** and CoronaVac (n=25/group) **(B, D)** vaccinations. Data shown are median and each symbol represents an individual. ns: not significant; *P<0.05.

A total of 110 participants (50% with type 2 diabetes) were recruited in Part 1 of this study, which evaluated the impact of type 2 diabetes on immunological memory to SARS-CoV2 at 3-6 months after two doses of BNT162b2 (n=60) or CoronaVac vaccinations (n=50) (**Supplementary Figure 1**). **Table 1** summarizes their baseline characteristics. Among both BNT162b2 and CoronaVac vaccinees, those with type 2 diabetes had significantly higher BMI than HCs. Otherwise, the baseline clinical characteristics were similar between BNT162b2 and CoronaVac recipients in both groups with or without type 2 diabetes. In serological analysis, the time of sampling after vaccination was inversely correlated with anti-RBD IgG titres among BNT162b2, but not CoronaVac, vaccinees (**Supplementary Figure 3**). Notably, CoronaVac vaccinees had significantly lower levels of anti-RBD IgG antibody as compared to those who received BNT162b2 (**Table 1**). Importantly, while seroconversion occurred in all BNT162b2 vaccinees with comparable anti-RBD IgG antibody titres between participants with type 2 diabetes and HCs (**Table 1**; **Figure 1A**), sera anti-RBD IgG was detected in only 88% and 92% of the CoronaVac vaccinees with or without type 2 diabetes, respectively. Among the CoronaVac vaccinees, the titres of anti-RBD IgG antibody were also lower in those with type 2 diabetes than HCs (**Table 1**), although this difference failed to reach statistical significance after adjustments for sex and BMI. (**Figure 1B**). We further examined the levels of neutralization antibodies, which provide a functional readout for vaccine-elicited humoral responses. Similarly, the levels of neutralizing antibodies induced by BNT162b2 were comparable between participants with type 2 diabetes and HCs (**Table 1**; **Figure 1C**). However, among CoronaVac vaccinees, the virus-neutralizing capacity of antibodies was significantly reduced in patients with type 2 diabetes even with adjustments for sex and BMI (p=0.017) (**Figure 1D**). In subgroup analyses within BNT162b2 or CoronaVac recipients who had type 2 diabetes, their levels of anti-RBD IgG and neutralizing antibodies were similar between those who had HbA1c above or below 7% (**Supplementary Figure 4**).

**Table 1:** Baseline characteristics of the participants in Part 1 of the study (N=110).

	BNT162B2			CoronaVac			T2D patients	HC
	T2D patients	HC	T2D vs HC p-value	T2D patients	HC	T2D vs HC p-value	BNT162B2 vs CoronaVac p-value	BNT162B2 vs CoronaVac p-value
N	30	30	--	25	25	--	--	--
Sex (men)	16	16	--	18	11	0.085	0.177	0.591
Age, years	53 (47–60)	44 (41-58)	0.061	57 (54-61)	55 (48-59)	0.321	0.095	0.060
BMI, kg/m2	26.8±3.0	23.2±2.6	**0.001**	27.8±5.0	24.1±3.5	**0.004**	0.416	0.502
Time since vaccine, weeks	17.1 (15.4-20.1)	20.9 (17.0-22.6)	**0.023**	17.0 (14.8-19.3)	18.3 (16.1-21.0)	0.174	0.483	0.074
Duration of diabetes, years	14.2±7.6	--	--	13.8±10.7	--	--	0.886	--
Hypertension	21 (70.0%)	--	--	18 (72.0%)	--	--	0.871	--
Dyslipidaemia	23 (76.7%)	--	--	21 (84.0%)	--	--	0.498	--
CAD	2 (6.7%)	--	--	5 (20.0%)	--	--	0.226	--
Stroke	0 (0%)	--	--	0 (0%)	--	--	--	--
OAD	30 (100%)	--	--	25 (100%)	--	--	--	--
Insulin	12 (40.0%)	--	--	8 (32.0%)	--	--	0.539	--
HbA1c, %	6.9 (6.5-7.2)	--	--	6.9 (6.7-7.5)	--	--	0.388	--
HbA1c ≥7%	13 (43.3%)	--	--	12 (48.0%)	--	--	0.729	--
HbA1c ≥8%	2 (6.7%)	--	--	3 (12.0%)	--	--	0.650	--
eGFR <60ml/min/1.73m2	2 (6.7%)	--	--	3 (12.0%)	--	--	0.650	--

Mean±standard deviation or median with interquartile range. T2D, type 2 diabetes; HC, healthy controls; BMI, body mass index; Ab, antibody; Neu, neutralization; CAD, coronary artery disease; OAD, oral anti-diabetic drugs; HbA1c, glycated haemoglobin; eGFR, estimated glomerular filtration rate. The bold values indicate p-values <0.05.

### Memory CD4^+^T immunity elicited by CoronaVac, but not BNT162b2, was affected by type 2 diabetes

**Figure 2:**
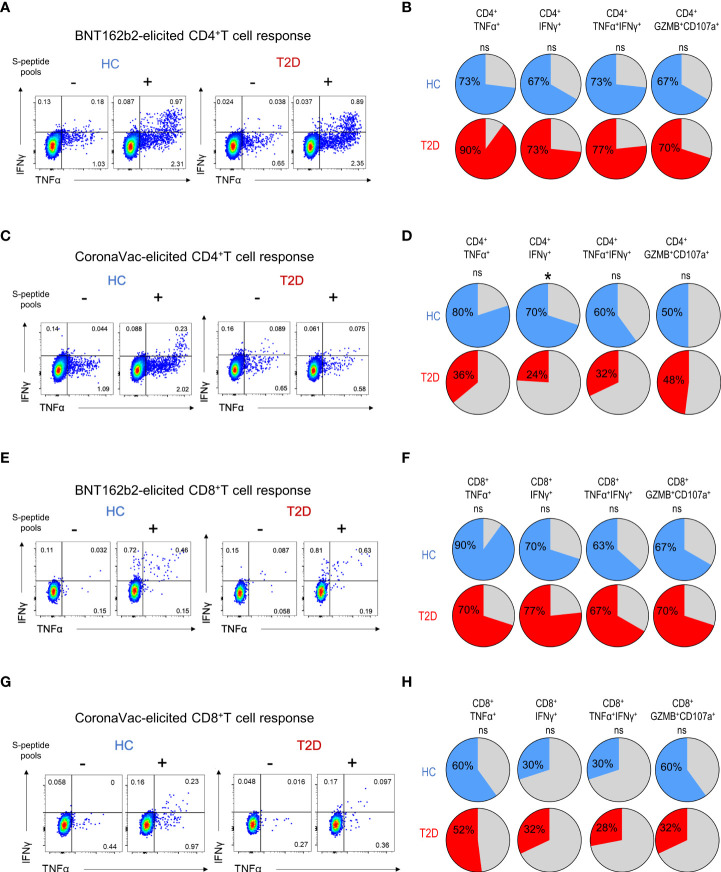
Proportions of participants harbouring memory T cell responses 3-6 months following BNT162b2 and CoronaVac vaccination. PBMCs collected from BNT162b2 (n=30/group) **(A, B, E, F)** and CoronaVac (n=10-25/group) **(C, D, G, H)** vaccinees were stimulated with S1 peptide pools. **(A, D)** Representative flow cytometry plots of cytokine-producing CD4^+^T cells with or without peptide stimulation. **(B, D)** Proportions of HCs and type 2 diabetic patients with cytokine (TNFα^+^, IFNγ^+^, TNFα^+^IFNγ^+^ and GZMB^+^CD107a^+^)-producing CD4^+^T cells following peptide stimulation. **(E, G)** Representative flow cytometry plots of cytokine-producing CD8^+^T cells with or without peptide stimulation. **(F, H)** Proportions of HCs and type 2 diabetic patients with cytokine (TNFα^+^, IFNγ^+^, TNFα^+^IFNγ^+^ and GZMB^+^CD107a^+^)-producing CD8^+^T cells following peptide stimulation. ns: not significant; *P<0.05.

**Figure 3:**
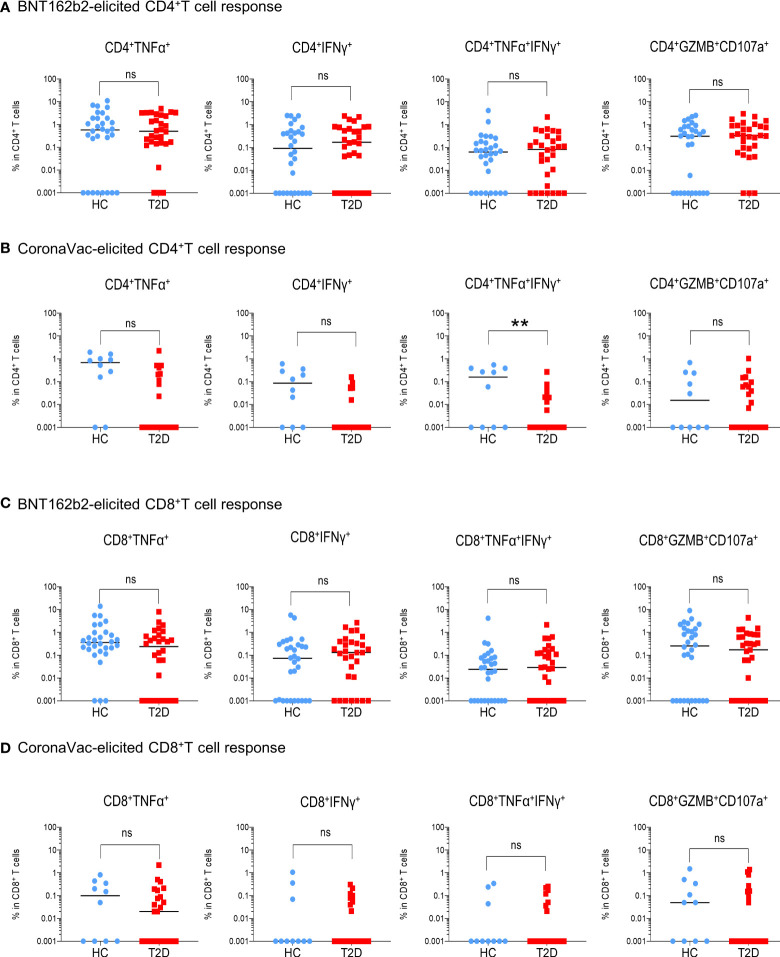
Extent of memory T cell responses 3-6 months following BNT162b2 and CoronaVac vaccination. PBMCs collected from BNT162b2 (n=30/group) **(A, C)** and CoronaVac (n=10-25/group) **(B, D)** vaccinees were stimulated with S1 peptide pools. **(A, D)** Percentages of cytokine (TNFα^+^, IFNγ^+^, TNFα^+^IFNγ^+^ and GZMB^+^CD107a^+^)-producing CD4^+^ **(A, B)** and CD8^+^ **(C, D)** T cells were determined by FACs analysis. Data shown are median and each symbol represents an individual. Statistical analysis were performed among the responders. ns: not significant; **P<0.01..

Next, we examined the presence of memory T cell immunity following BNT162b2 vaccination. Positive CD4^+^T cell response was defined as the presence of TNFα and/or IFNγ production following peptide stimulation and similar responses were observed between participants with type 2 diabetes and HCs (**Figure 2A**). Most participants (67-73%) had detectable circulating S-reactive CD4^+^T cells with an increase reactivity to S2 peptide pool (**Figure 2B**; **Supplementary Figure 5A**). Moreover, similar frequencies of CD4^+^T cells that produced individual cytokines or co-produced TNFα and IFNγ following S1 or S2 peptide stimulation were detected in responders with or without type 2 diabetes (**Figure 3A**; **Supplementary Figure 6A**). In addition, the proportion of S-reactive CD4^+^T cells with cytotoxic potential (GZMB^+^CD107a^+^) was also comparable between participants with type 2 diabetes and HCs (**Figures 2B**, **3A**; **Supplementary Figures 5A**, **6A**). In contrast, among the CoronaVac vaccinees, only 60-80% of HCs had detectable circulating S-reactive CD4^+^T cells following S1 peptide stimulation, and this proportion further reduced to 24-36% in participants with type 2 diabetes. Specifically, the proportion of CoronaVac vaccinees harbouring S1-reactive IFNγ^+^CD4^+^T cells was significantly reduced in participants with type 2 diabetes (p=0.02) after adjustment for BMI. (**Figures 2C, D**). Furthermore, among those with detectable S1-reactive CD4^+^T cell responses, the frequency of polyfunctional CD4^+^T cells which co-produced IFNγ and TNFα was also significantly lower in participants with type 2 diabetes (p=0.005) (**Figure 3B**). Less reactivity to S2 peptide pools were observed in HCs, with only 40-50% showing positive CD4^+^T cell response and this rate remained similar in participants with type 2 diabetes (**Supplementary Figures S5B**; **6B**).

For memory CD8^+^T cell immunity, the majority of BNT162b2 vaccinees (63-90%) had detectable S-reactive CD8^+^T cells with similar reactivity towards both S1 and S2 peptides (**Figures 2E, F** **;** **Supplementary Figure 5C**). Similar to CD4^+^T cell memory responses, the proportions of participants bearing S-reactive CD8^+^T cells and the frequencies of cytokine-producing and cytotoxic CD8^+^T cells among the responders were comparable between HCs and participants with type 2 diabetes (**Figures 2F**, **3C** **;** **Supplementary Figures S5C**, **6C**). In contrast, only 30-60% of the CoronaVac vaccinees had detectable circulating S-reactive CD8^+^T cells (**Figures 2G, H**; **Supplementary Figure 5D**). Furthermore, polyfunctional CD8^+^T cells, as indicated by their ability to co-produce TNFα and IFNγ, were also less abundant in CoronaVac vaccinees compared with those who received BNT162b2 (**Figures 3C, D** **;** **Supplementary Figures S6C, D**). Nonetheless, the presence of type 2 diabetes did not lead to further reduction in the frequencies of cytokine-producing and cytotoxic CD8^+^T cells elicited by CoronaVac (**Figure 3D**; **Supplementary Figure 6D**).

### Initial induction of S-reactive CD4^+^T cells by BNT162b2 was attenuated in patients with type 2 diabetes

Thus far, our findings demonstrated that the immunological memory elicited by two doses of BNT162b2 was well-maintained in patients with type 2 diabetes, mirroring the clinical performance of this vaccine reported in other studies (30–33). Notably, a recent study reported that patients with type 2 diabetes still suffer from more severe COVID-19 symptoms when only one dose of BNT162b2 was administrated (31). To gain mechanistic insights on the initial B and T cell responses induced by BNT162b2, a total of 16 BNT162b2 vaccinees (50% with type 2 diabetes) were further recruited to Part 2 of this study (**Supplementary Figure 2**), with their baseline characteristics summarised in **Table 2**.

**Table 2:** Baseline characteristics of the participants in Part 2 of the study (N=16).

	T2D patients	Healthy controls	p-value
N	8	8	--
Men	2 (25%)	2 (25%)	1.00
Age, years	58 (49-63)	56 (42-59)	0.328
BMI, kg/m2	28 (25-34)	22 (19-25)	**0.007**
Duration of diabetes, years	13 (6-23)	--	--
Hypertension	6 (75%)	--	--
Dyslipidaemia	6 (75%)	--	--
CAD	0	--	--
Stroke	0	--	--
OAD	8 (100%)	--	--
Insulin	4 (50%)	--	--
HbA1c, %	7.3 (6.8-7.6)	--	--
HbA1c ≥7%	5 (62.5%)	--	--
HbA1c ≥8%	0	--	--
eGFR <60ml/min/1.73m2	1 (12.5%)	--	--

T2D, type 2 diabetes; BMI, body mass index; CAD, coronary artery disease; OAD, oral antidiabetic drugs; HbA1c, glycated haemoglobin; eGFR, estimated glomerular filtration rate. The bold values indicate p-values <0.05.

**Figure 4:**
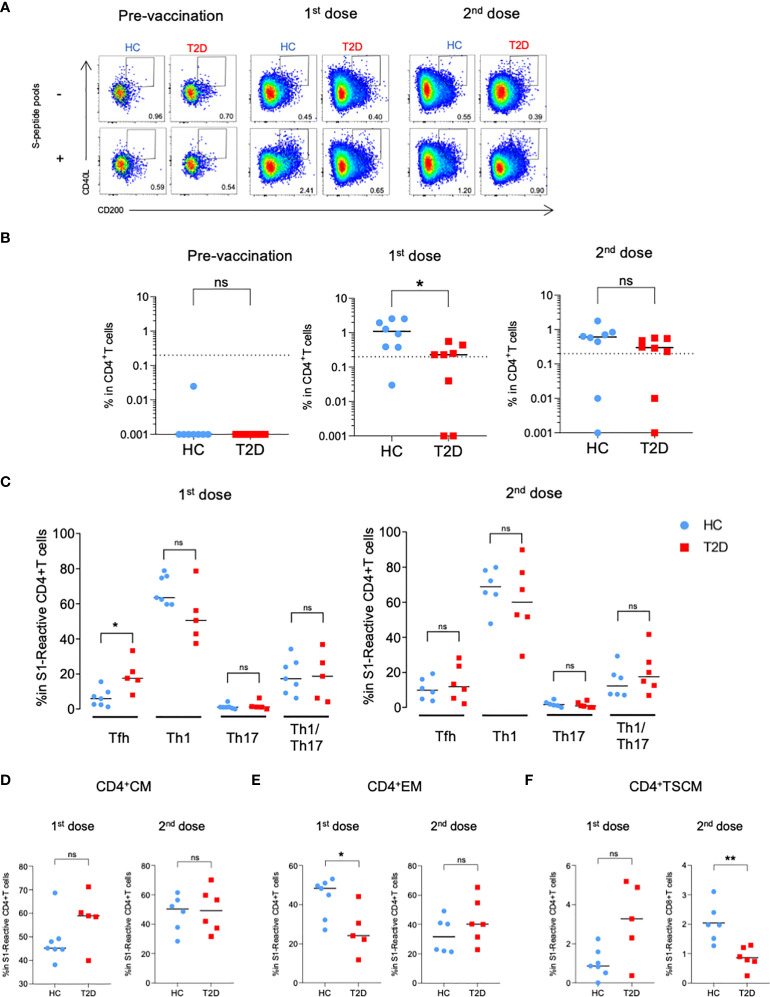
S1-reactive CD4^+^T Cell responses shortly after BNT162b2 vaccination. A cohort of HCs and type 2 diabetic patients (n=8/group) were recruited for time course analysis of immune responses following BNT162b2 vaccination. PBMCs collected at pre-vaccination, 20 days after the 1^st^ dose and 14-20 days after the 2^nd^ dose of injections were stimulated with S1 peptide pools. **(A)** Representative flow cytometry plots depicting the gating of CD40L^+^CD200^+^ (gating) to identify antigen-reactive CD4^+^ cells following peptide stimulation. Dotted line represents the limit of detection. **(B)** Proportions of S1-reactive CD4^+^ T cells. **(C)** Proportions of CD4^+^T cell subsets in S1-reactive CD4^+^ T cells (n=5-7/group). Tfh, CXCR5^+^; Th1, CXCR5^-^CXCR3^+^CCR6^-^; Th17, CXCR5^-^CCR6^+^; Th1/17, CXCR5^-^CXCR3^+^CCR6^+^. **(D-F)** Proportions of memory T cell subsets in S1-reactive CD4^+^ T cells(n=5-7/group). **(D)** central memory (CM), CD45RA^-^ CCR7^+^. **(E)** effector memory (EM), CD45RA^-^CCR7^-^. **(F)** T stem cell memory (TSCM), CCR7^+^CD45RA^+^CD27^+^CD95^+^. Data shown are median and each symbol represents an individual **(B-F)**. ns: not significant; *P<0.05.

The levels of anti-RBD IgG antibody were apparently lower in participants with type 2 diabetes as compared to HCs following their first dose of BNT162b2. However, the difference was not statistically significant and the levels became comparable after the second dose (**Supplementary Figure 7**). When evaluating the BNT162b2-elicited cellular immunity, we observed a rapid induction of activated CD4^+^T cells responding to S1 peptide pools in all HCs following their first dose of BNT162b2, and this response did not further increase after the second dose (**Figures 4A, B**). However, among participants with type 2 diabetes, the proportion of S1-reactive CD4^+^T cells following the first dose was significantly lower compared with HCs, and this reduction was rectified after the second dose (**Figure 4B**). Similar trend of reduction in the proportion of S2-reactive CD4^+^ cells was also observed in participants with type 2 diabetes after the first dose, although this difference did not reach statistical significance (**Supplementary Figure 8A**).

### S-reactive CD4^+^T cells with reduced Th1 differentiation were found in participants with type 2 diabetes

We then examined the subset differentiation of S-reactive CD4^+^T cells in the BNT162b2-vaccinated individuals. In HCs, S1-reactive CD4^+^T cells were predominantly characterised by Th1 phenotype, followed by Tfh and Th1/17, and finally Th17 phenotypes, which collectively indicated an anti-viral centric response (**Figure 4C**). Of note, a trend of reduction of Th1 cells with a reciprocal increase in Tfh cells were observed in participants with type 2 diabetes after the first dose, although these differences were rectified after the second dose (**Figure 4C**). Similar changes in the percentage of Th1 cells was also detected in S2-reactive CD4^+^T cells from participants with type 2 diabetes (**Supplementary Figure 8B**). Consistently, S-reactive CD4^+^T cells in participants with type 2 diabetes exhibited significantly lower expression of IFNγ, the defining cytokine for Th1 response following the first dose (**Supplementary Figure 9**). Notably, all these T cell response parameters were not associated with BMI, except for S2-reactive Th1 cells. Nonetheless, in quantile regression analysis, the proportion of S2-reactive Th1 cells remained significantly lower in participants with type 2 diabetes than HCs (p=0.049) after BMI adjustment.

### BNT162b2 generated T stem cell memory (TSCM) cells in both HCs and participants with type 2 diabetes

Within the BNT162b2-elicited S-reactive CD4^+^T cells, the proportions of CM and EM cells were largely comparable between HCs and participants with type 2 diabetes following both doses of vaccination (**Figures 4D, E**; **Supplementary Figure 8C**). Increases in the percentage of S-reactive CD4^+^ TSCM cells were observed in participants with type 2 diabetes after their first dose of BNT162b2, but this difference was no longer observed after the second dose (**Figure 4F**; **Supplementary Figure 8C**).

### Initial BNT162b2-elicited CD8^+^T cell responses were comparable between HCs and participants with type 2 diabetes

**Figure 5:**
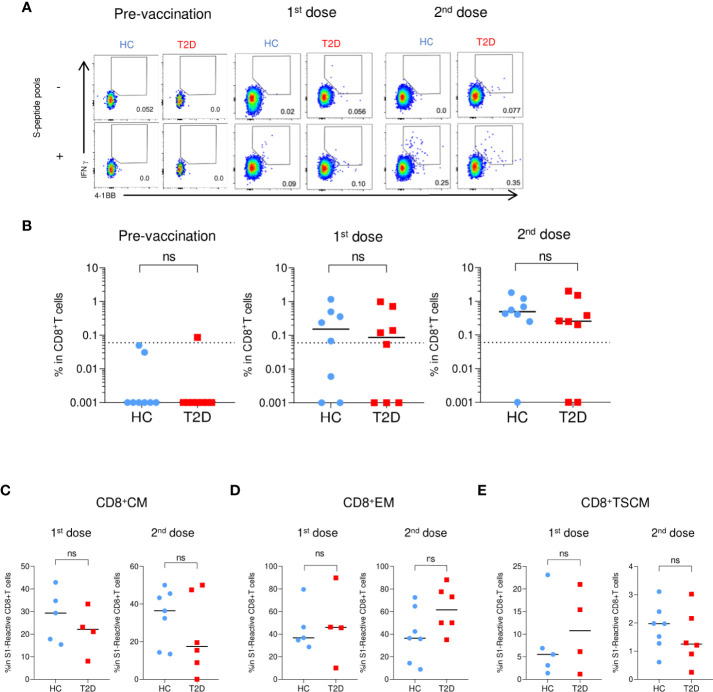
S1-reactive CD8^+^T Cell responses shortly after BNT162b2 vaccination. PBMCs collected from BNT162b2 vaccinees for time course analysis were stimulated with S1 peptide pools (n=8/group). **(A)** Representative flow cytometry plots depicting the gating of IFNγ^+^4-1BB^+^ (gating) to identify antigen-specific S1-reactive CD8^+^ cells following peptide stimulation. Dotted line represents the limit of detection. **(B)** Proportions of S1-reactive CD8^+^ T cells. **(C-E)** Proportions of memory T cell subsets in S1-reactive CD8^+^ T cells (n=4-7/group). **(C)** central memory (CM), CD45RA^-^ CCR7^+^. **(D)** effector memory (EM), CD45RA^-^CCR7^-^. **(E)** T stem cell memory (TSCM), CCR7^+^CD45RA^+^CD27^+^CD95^+^. Data shown are median and each symbol represents an individual (n=4-7/group) **(B-E)**. ns: not significant.

Regarding the S-reactive CD8^+^T cell responses, modest numbers of activated CD8^+^T cells responding to S1 and S2 peptide pools were detected after the first dose of BNT162b2, and their proportions further expanded by two- to five-fold following the second dose (**Figures 5A, B** **;** **Supplementary Figure 10A**). In contrast to the S-reactive CD4^+^T cell responses, the proportions of S1- and S2-reactive CD8^+^ T cells in HCs and type 2 diabetes were comparable following both doses of vaccination. (**Figure 5B**; **Supplementary Figure 10A**). We then evaluated the memory subsets distribution among S-reactive CD8^+^T cells. No detectable differences were observed in the CM and EM subsets between HCs and participants with type 2 diabetes following both doses of vaccination (**Figures 5C, D** **;** **Supplementary Figures S10B**). However, in contrast to the findings with CD4^+^TSCM, comparable level of S1-reactive CD8^+^TSCM was detected in HCs and participants with type 2 diabetes after both doses (**Figure 5E**). Although lower levels of S2-reactive CD8^+^TSCM was observed in participants with type 2 diabetes following their second dose (**Supplementary Figure 10B**), this reduction did not affect the proportion of S2-reactive CD8^+^T cells at 3-6 months after vaccination (**Supplementary Figures S5C**, **6C**).

## Discussion

The impact of type 2 diabetes on immune responses elicited by the different COVID-19 vaccines remains undefined. In this study, we compared the induction and longevity of both cellular and humoral immunity induced by mRNA and inactivated COVID-19 vaccines between HCs and patients with type 2 diabetes. Our findings demonstrated that mRNA vaccine (BNT162b2) effectively induced antigen-specific CD4^+^ and CD8^+^ memory T cells, as well as anti-RBD IgGs and neutralizing antibodies in all vaccinees regardless of the presence of type 2 diabetes. However, a defect in the initial CD4^+^T cell response, exemplified by reductions in S-reactive CD4^+^T cells and Th1 differentiation, was found in patients with type 2 diabetes before their second dose of vaccination. CD4^+^T cells consist of multiple subsets with distinct functions. Given the importance of CD4^+^Th1 cells in anti-viral responses, our findings have provided a potential mechanism to explain the existing clinical observations showing worse COVID-19 outcomes in patients with type 2 diabetes who only received a single dose of BNT162b2 (30–33). The durability of protective immunity is conferred by the induction of memory T cells, particularly TSCM cells, which represent a subset of long-lived T cells with robust self-renewal capacity (34). Of note, patients with type 2 diabetes showed an initial increment of CD4^+^TSCM, which may explain their comparable memory T cell responses and similar protection against severe COVID-19 infection as HCs following the second dose of BNT162b2 (25).

On the other hand, we demonstrated that the humoral response was reduced in patients with type 2 diabetes despite receiving two doses of inactivated vaccine (CoronaVac). This could explain their worse clinical performance with breakthrough infections (35). Although the levels of anti-RBD IgG antibodies were comparable between HCs and patients with type 2 diabetes, the levels of functional neutralizing antibodies elicited by CoronaVac were significantly reduced in patients with type 2 diabetes after adjustments for potential confounders including BMI. Patients with type 2 diabetes are known to have elevated levels of glycosylated IgGs that could lower the affinity of an IgG for its antigen (36–39). We speculate that this effect might have a more significant impact on the neutralization capacity of anti-RBD IgGs when their levels are low, as observed in the CoronaVac vaccinees, but is less relevant when IgG levels are high as in the BNT162b2 vaccinees.

In this study, PBMCs were only available from 35 CoronaVac vaccinees (10 HCs and 25 with type 2 diabetes), which has limited more in-depth examination of the CoronaVac-elicited T cell responses. Nonetheless, a preliminary analysis using these 35 PBMCs samples still showed that CoronaVac-elicited cellular memory responses were impaired in patients with type 2 diabetes. The mechanisms underlying the poor response to inactivated vaccines in patients with type 2 diabetes remain to be elucidated. CD4^+^T cells are crucial in ensuring effective responses by other lymphocytes, including B cells for optimised antibody production (40). Given that an impaired CD4^+^T cell differentiation was detected after the first dose of BNT162b2 vaccination, it is possible that the poor vaccine efficacy with CoronaVac among patients with type 2 diabetes is also related to a compromised initial CD4^+^T cell response. However, more in-depth analysis of the earlier cellular immune reaction to CoronaVac vaccination is required to investigate this hypothesis.

Emerging data suggest that anti-RBD antibodies elicited by COVID-19 vaccines wane rapidly and are less effective in neutralizing the fast-spreading Omicron variant (41–43). However, vaccine-elicited S-reactive T cell responses are durable with extensive cross-reactivity against Omicron variant (44) highlighting the importance of these memory T cells in conferring protection against current and emerging SARS-CoV2 variants. In addition, CD4^+^T cell differentiation during acute SARS-CoV2 infection is a key factor that determines clinical outcome. While the development of an anti-viral Th1 phenotype is essential in combating viral infection, Th2- and Th17-skewed differentiation patterns are predictive of adverse outcomes (45, 46). In this study, we observed poorer antigen-specific Th1 response in patients with type 2 diabetes following a single dose of mRNA vaccine but not after the second dose. This reflected the clinical performance of BNT162b2 vaccine in patients with type 2 diabetes showing that the efficacy of the initial dose was impaired (31) until a booster shot was administered (30–33). Glucose metabolism and T cell differentiation are heavily intertwined. Insulin resistance, which is a hallmark of type 2 diabetes, might contribute to T cell dysfunction. Impaired glycolysis and mitochondrial respiration have been shown to be present in T cells of patients with type 2 diabetes (9). Given the reliance of Th1 differentiation on glycolysis (47), the CD4^+^T cell defect in patients with type 2 diabetes could occur as a consequence of a reduction in glucose uptake. Indeed, impaired insulin signalling has been shown to hinder the uptake of glucose in T cells, thus providing a potential mechanism for such defects in type 2 diabetes (48). Interestingly, our findings suggested that these defects can be rectified by a more potent vaccine regimen (e.g. double inoculation of mRNA vaccines), although more extensive clinical and animal studies with appropriate models are required to fully address this issue in the future.

Our study has several limitations, including the small sample size, exclusion of elderly individuals and the long-term T cell stimulation assay which could have potentially introduced bias for high avidity clones. Follow-up studies involving a larger cohort of patients with type 2 diabetes covering a wider spectrum of comorbidities, preferably with a complete sex-, age- and BMI-matched control cohort, are required to validate our findings. Furthermore, we have not been able to address whether poor glycaemic control affects the immune response as the number of patients with HbA1c >8% was small. We have also not evaluated the immune response after a second booster dose as it was not available at the time our study was carried out.

## Conclusion

Overall, type 2 diabetes impaired both cellular and humoral immune responses induced by the inactivated vaccine. However, the mRNA vaccine response in patients with type 2 diabetes is reassuring. Although there was a defect in the initial CD4^+^T cell Th1 differentiation, this impairment could be overcome by the administration of a booster mRNA vaccine dose. The findings from this mechanistic study suggest that patients with type 2 diabetes who received mRNA vaccines can achieve similar cellular and humoral immunity as seen in HCs. Despite the ongoing COVID-19 pandemic and recommendations from healthcare institutions and governing bodies, vaccination is still entirely voluntary and the uptake of COVID-19 vaccines remains unsatisfactory in many places. Our findings reinforce the need for wide-spread vaccination and help inform stakeholders when providing vaccine recommendations, particularly to individuals with type 2 diabetes.

## Data availability statement

The original contributions presented in the study are included in the article/**Supplementary Material**. Further inquiries can be directed to the corresponding authors.

## Ethics statement

The studies involving human participants were reviewed and approved by Institutional Review Board (IRB) of the University of Hong Kong/Hospital Authority Hong Kong West Cluster (IRB Ref: UW 21-460). The patients/participants provided their written informed consent to participate in this study.

## Author contributions

C-HL, VG, and GL collected and analysed the data, and wrote the manuscript. JT, DT-WL, AT, and PP collected and analysed the data. VG and C-YF performed statistical analyses. GL, I-NH, and K-BT critically reviewed and edited the manuscript. GL, I-NH, and K-BT initiated and supervised the study, and were the guarantors of this work and as such had full access to all the data in the study and take responsibility for the integrity of the data and the accuracy of the data analysis. All authors contributed to the article and approved the submitted version.

## Funding

This study was supported in part by the Health and Medical Research Fund (Ref. COVID1903010) and from an Endowment Fund established for the “Sir David Todd Professorship in Medicine” awarded to K-BT.

## Acknowledgments

We thank all the participants who contributed to this study.

## Conflict of interest

The authors declare that the research was conducted in the absence of any commercial or financial relationships that could be construed as a potential conflict of interest.

## Publisher’s note

All claims expressed in this article are solely those of the authors and do not necessarily represent those of their affiliated organizations, or those of the publisher, the editors and the reviewers. Any product that may be evaluated in this article, or claim that may be made by its manufacturer, is not guaranteed or endorsed by the publisher.

## Supplementary material

Supplementary Figure 1 Schematic diagram of the study

Supplementary Figure 2 Gating strategy for T cell analysis. **(A)** Gating of viable single T cells. **(B)** Gating of cytokine (TNFα^+^, IFNγ^+^ and TNFα^+^IFNγ^+^) producing CD4^+^(left) and cells CD8^+^ (right). **(C)** Gating of S-reactive CD4^+^ (CD220^+^CD40L^+^) (left) and S-reactive CD8^+^ (IFNγ^+^4-1BB^+^) (right) T cells. **(D)** Characterization of Th subsets among S-reactive CD4^+^T cells. **(E)** Characterization of memory subsets among S-reactive CD4^+^T cells. **(F)** Characterization of memory subsets among S-reactive CD8^+^T cells. Red dots represent S-reactive cells overlaid on total CD4^+^ or CD8^+^ cells (grey contour plots with outliers). Numbers inside or adjacent to the gates indicate frequencies within S-reactive T cells. Isotype-matched control antibodies were used to determine the specificity of the staining and the gating boundaries.

Supplementary Figure 3 Correlations between time since vaccination and levels of anti-RBD IgG and neutralizing antibodies. Serum titres of anti-RBD IgG and neutralizing antibodies elicited by BNT162b2 (n=60) **(A, C)** and CoronaVac (n=50) **(B, D)** are plotted against the time of sampling after the second vaccine dose. Each symbol represents an individual. *P* values were derived from Spearman correlation.

Supplementary Figure 4 Levels of anti-RBD IgG and neutralizing antibodies in type 2 diabetic patients 3-6 months following BNT162b2 and CoronaVac vaccination Serum titres of anti-RBD IgG and neutralizing antibodies measured at 3-6 months following the second dose of BNT162b2 **(A, C)** and CoronaVac **(B, D)** vaccinations. Type 2 diabetic patients were divided into well-controlled (HbA1c<7%) and poor-controlled (HbA1c ≥ 7%) groups based on the degree of their glycaemic control. Serum anti-RBD IgG titres and neutralizing were measured 3-6 months following the second dose of BNT162b2 **(A)** and CoronaVac **(B)** vaccinations. Data shown are median and each symbol represents an individual. ns: not significant.

Supplementary Figure 5 Proportions of participants harbouring S2-reactive memory T cell responses 3-6 months following BNT162b2 and CoronaVac vaccination. PBMCs collected from BNT162b2 (n=30/group) **(A, C)** and CoronaVac (n=10-25/group) **(B, D)** vaccines were stimulated with S2 peptide pools. Proportions of HCs and type 2 diabetic patients with cytokine (TNFα^+^, IFNγ^+^, TNFα^+^IFNγ^+^ and GZMB^+^ CD107a^+^) producing CD4^+^ **(A, B)** and CD8^+^ **(C, D)** T cells following peptide stimulation. ns: not significant.

Supplementary Figure 6 Extent of S2-reactive memory T cell responses 3-6 months following BNT162b2 and CoronaVac vaccination. PBMCs collected from BNT162b2 (n=30/group) **(A, C)** and CoronaVac(n=10-25/group) **(B, D)** vaccines were stimulated with S2 peptide pools. Percentages of HCs and type 2 diabetic patients with cytokine (TNFα^+^, IFNγ^+^, TNFα^+^IFNγ^+^ and GZMB^+^ CD107a^+^) producing CD4^+^ **(A, B)** and CD8^+^ **(C, D)** T cells were determined by FACs analysis. Data shown are median and each symbol represents an individual. Statistical analysis were performed among the responders. ns: not significant.

Supplementary Figure 7 Levels of anti-RBD IgG antibodies shortly after BNT162b2 vaccination. A cohort of HCs and type 2 diabetic patients (n=8/group) were recruited from time course analysis of immune responses following BNT162b2 vaccination. Serum samples were collected before, 20 days after the 1st dose, and 14-20 days after the 2nd dose of injections. Anti-RBD IgG titres were measured by ELISA. Data shown are median. and each symbol represents an individual. ns: not significant.

Supplementary Figure 8 S2-reactive CD4^+^T Cell responses shortly after BNT162b2 vaccination. PBMCs collected from BNT162b2 vaccinees for time course analysis were stimulated with S2 peptide pools. **(A)** Frequency of S2-reactive CD4^+^ T cells. Dotted line represents the limit of detection. **(B)** Frequencies of CD4^+^T cell subsets in S2-reactive CD4^+^T cells. **(C)** Frequencies of memory T cell subsets in S2-reactive CD4^+^T cells. CM= central memory cells, EM= effector memory cells, TSCM = T stem cell memory cells. Data shown are median and each symbol represents an individual. ns: not significant; ***P<0.01*

Supplementary Figure 9 S-reactive IFNγ^+^CD4^+^T Cell responses shortly after BNT162b2 vaccination. PBMCs collected after 1^st^ and 2^nd^ dose of BNT162b2 vaccination were subjected to FACs analysis. Frequencies of IFNγ^+^cells in S-reactive CD4^+^T cells following S1 **(A)** and S2 **(B)** peptide pools stimulation. Data shown are median and each symbol represents an individual. ns: not significant; **P<0.05.*

Supplementary Figure 10 S2-reactive CD8^+^T Cell responses shortly after BNT162b2 vaccination. PBMCs collected from BNT162b2 vaccinees for time course analysis were stimulated with S2 peptide pools. **(A)** Frequency of S2-reactive CD8^+^ T cells. **(B)** Frequencies of memory T cell subsets in S2-reactive CD8^+^T cells. CM= central memory cells, EM= effector memory cells, TSCM = T stem cell memory cells. Data shown are median and each symbol represents an individual. ns: not significant; **P<0.05.*

## Abbreviations

CM, Central memory; COVID-19, Coronavirus disease 2019; EM, Effector memory; GZMB, Granzyme B; HC, Healthy controls; IRB, Institutional Review Board; PBMCs, Peripheral blood mononuclear cells; RBD, Receptor binding domain; S protein, Spike protein; SARS-CoV2, Severe acute respiratory syndrome coronavirus 2; Tfh, T-follicular-helper; Th, T-helper; TSCM, T stem cell memory.
