# Supplementary material for: Comparing the B and T cell-mediated immune responses in patients with type 2 diabetes receiving mRNA or inactivated COVID-19 vaccines
Source: Front Immunol. 2022 Oct 11;13:1018393. doi: 10.3389/fimmu.2022.1018393 (PMC9592994; doi:10.3389/fimmu.2022.1018393)
Supplement: Supplementary file 1 [file DataSheet_1.pdf]

Supplementary Figure 1

A

1st cohort:

BNT162b2-vaccinated

HC(n=30) vs T2D(n=30)

CoronaVac-vaccinated

HC(n=25*) vs T2D(n=25)

Blood collected at 3-6 months following 2nd dose of vaccination

Assessments:
(a) Humoral responses (anti-RBD-IgG titers ; neutralization capacity)
(b) Cellular responses (S-specific $CD4^+$ and $CD8^+$T cell analysis)

** Cellular responses were analyzed only in 10 HC participants*

B

2nd cohort:

HC(n=8) vs T2D(n=8)

BNT162b2 vaccination

Blood collected at :
(i) Before vaccination
(ii) 20 days after the first dose
(iii) 14-20 days after the second dose

Assessments:
(a) Humoral responses (anti-RBD-IgG titers)
(b) Cellular responses (S-specific $CD4^+$ and $CD8^+$T cell analysis)

Supplementary Figure 2

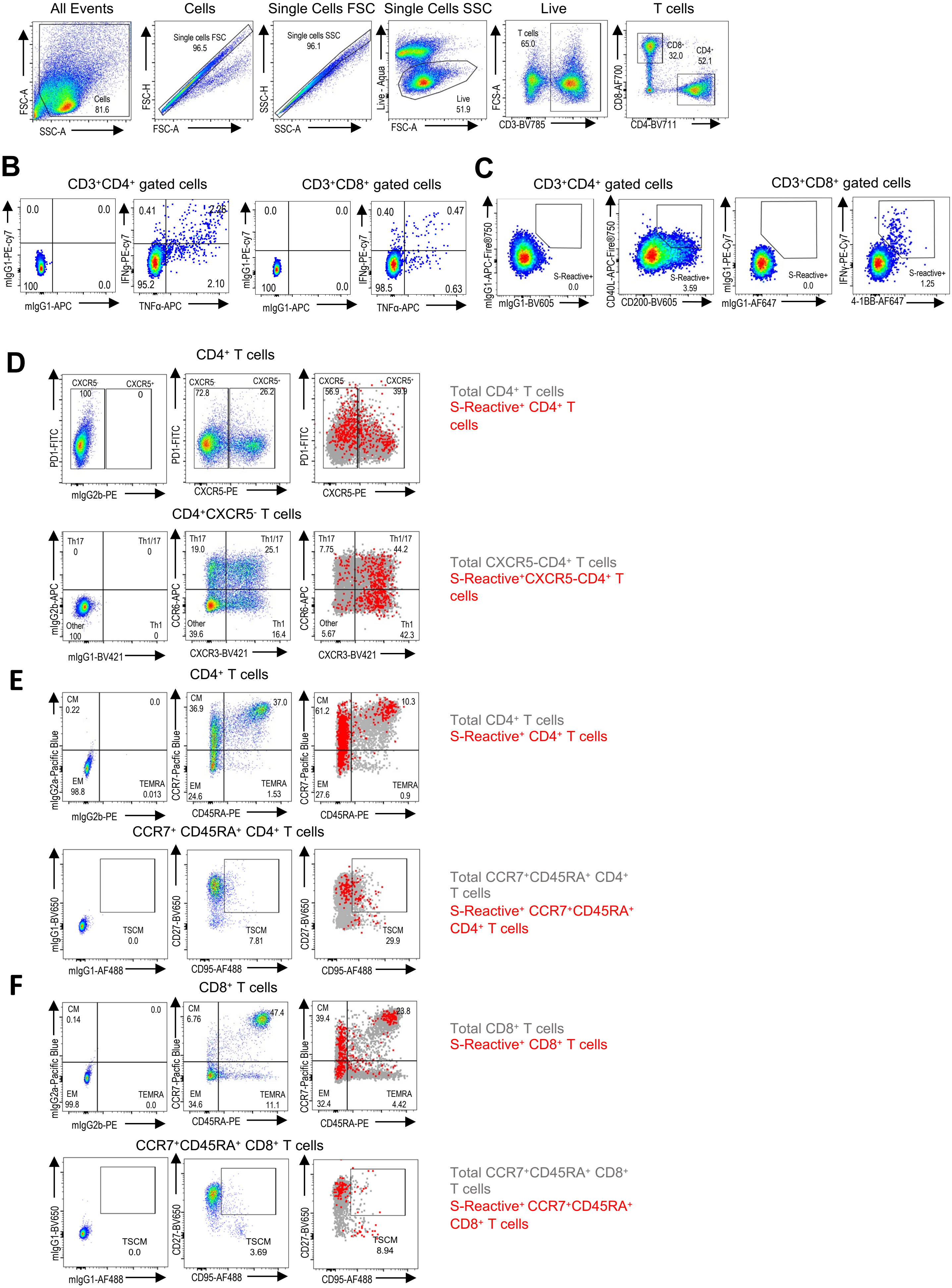

Supplementary Figure 3

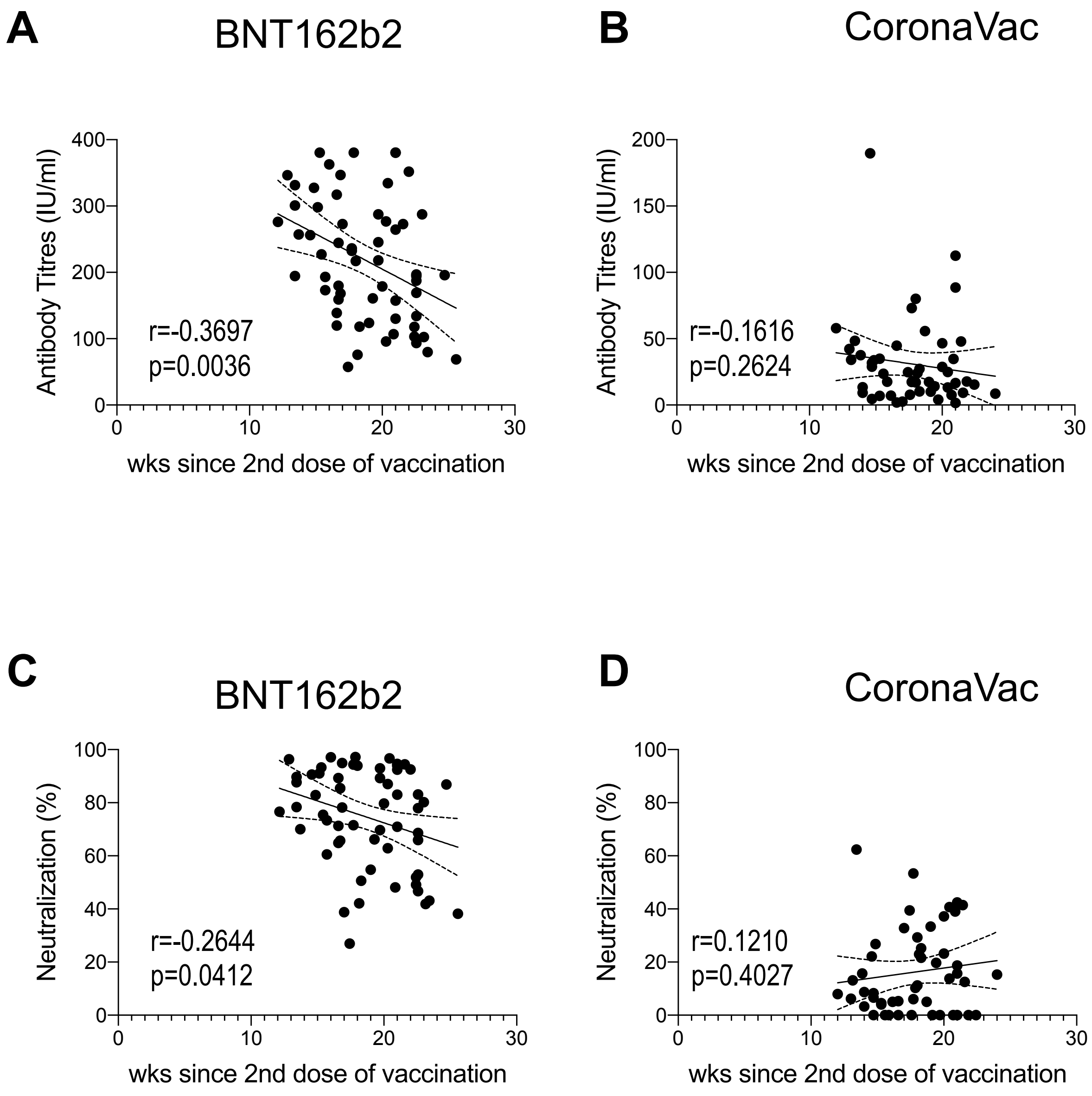

Supplementary Figure 4

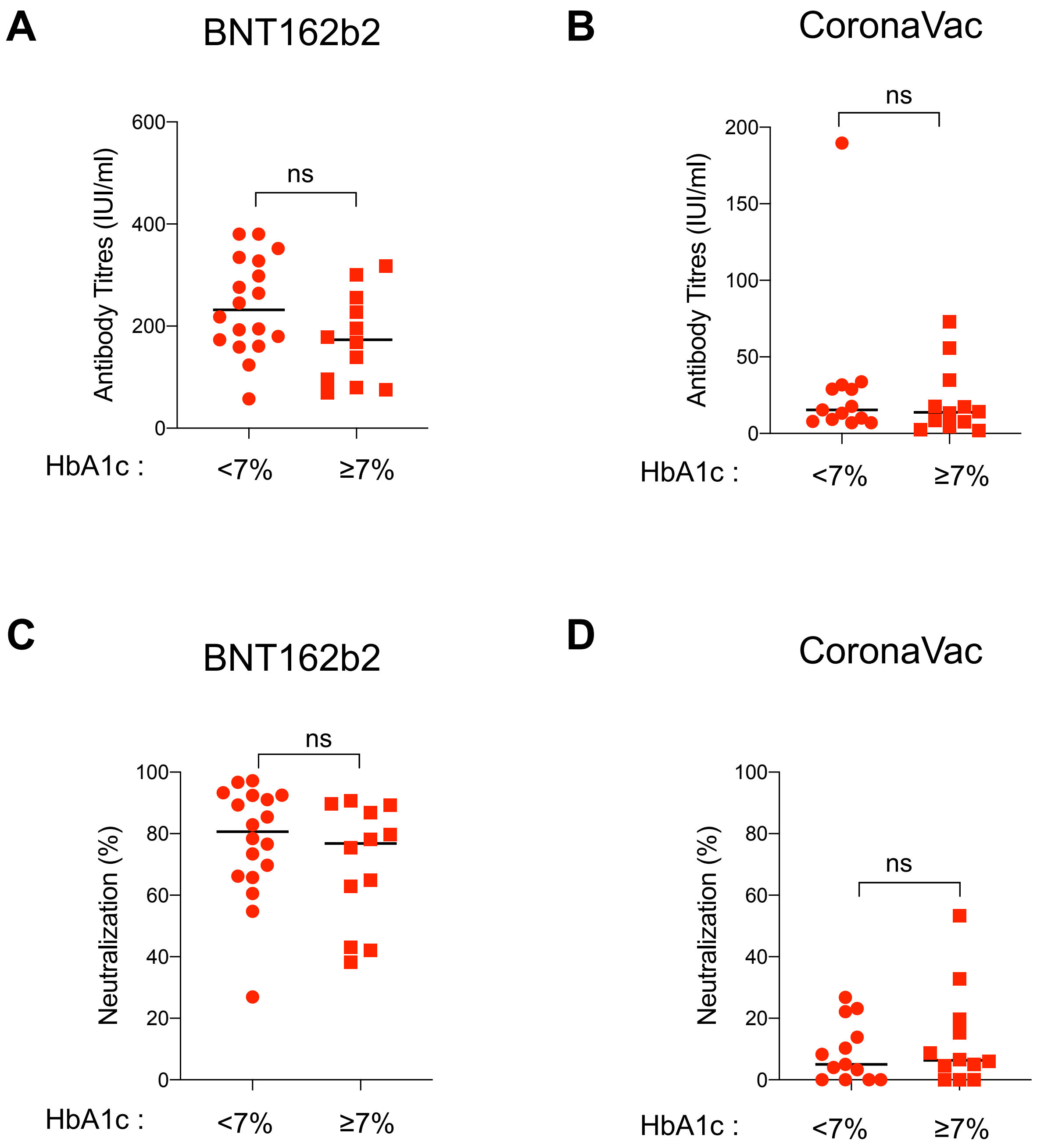

Supplementary Figure 5

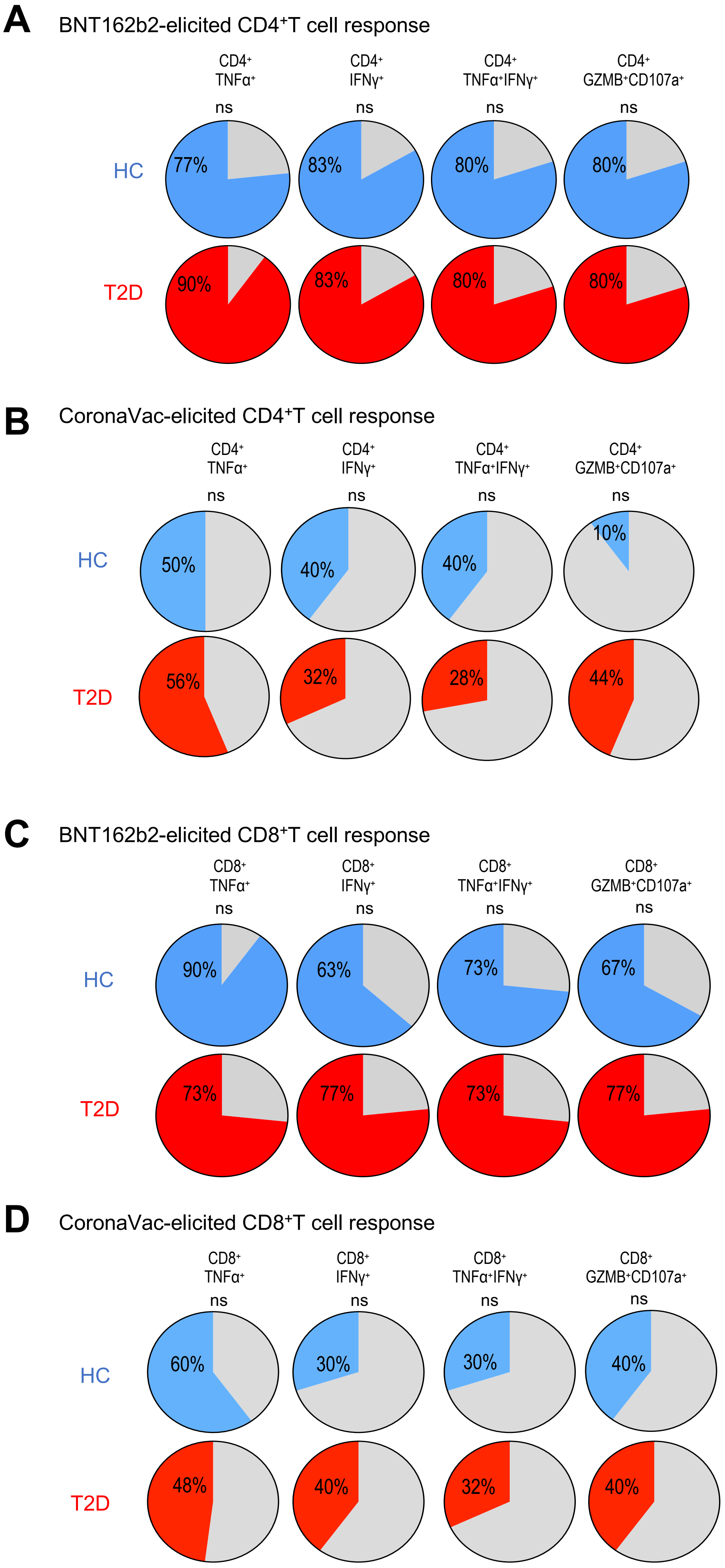

Supplementary Figure 6

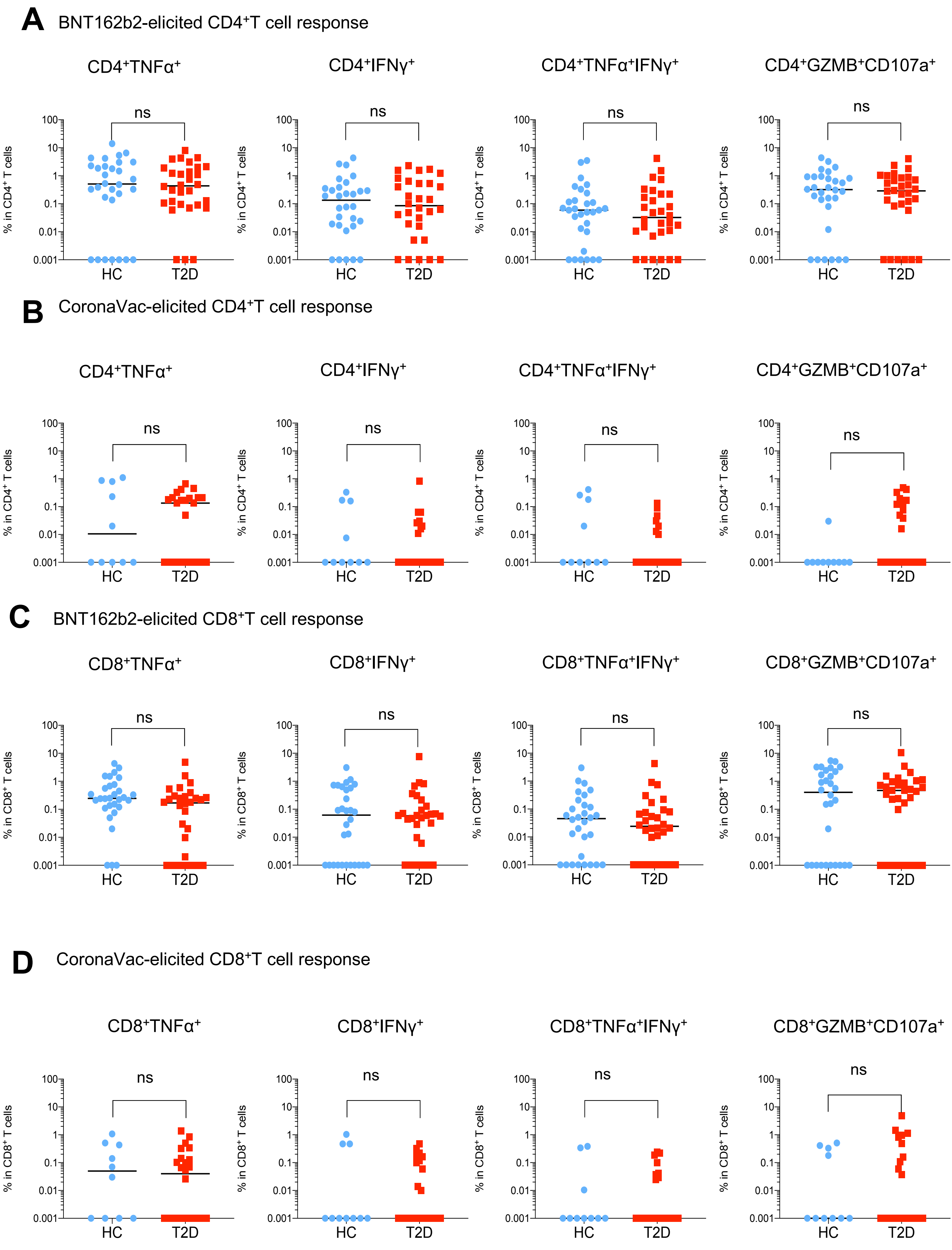

Supplementary Figure 7

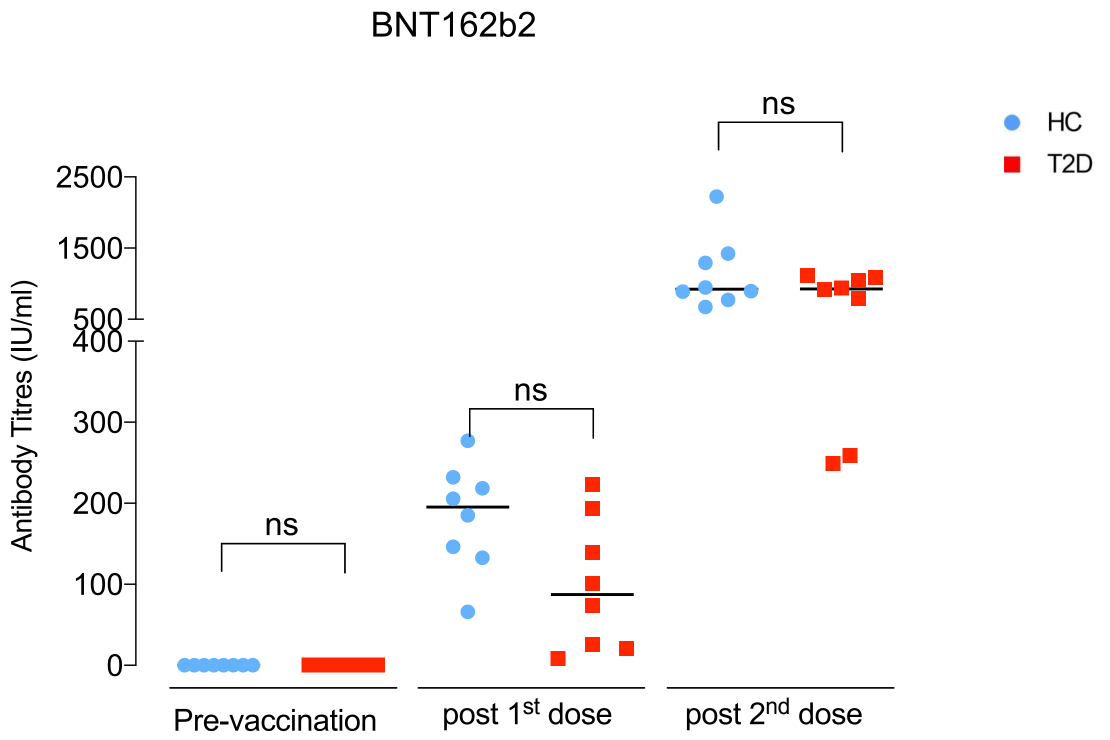

Supplementary Figure 8

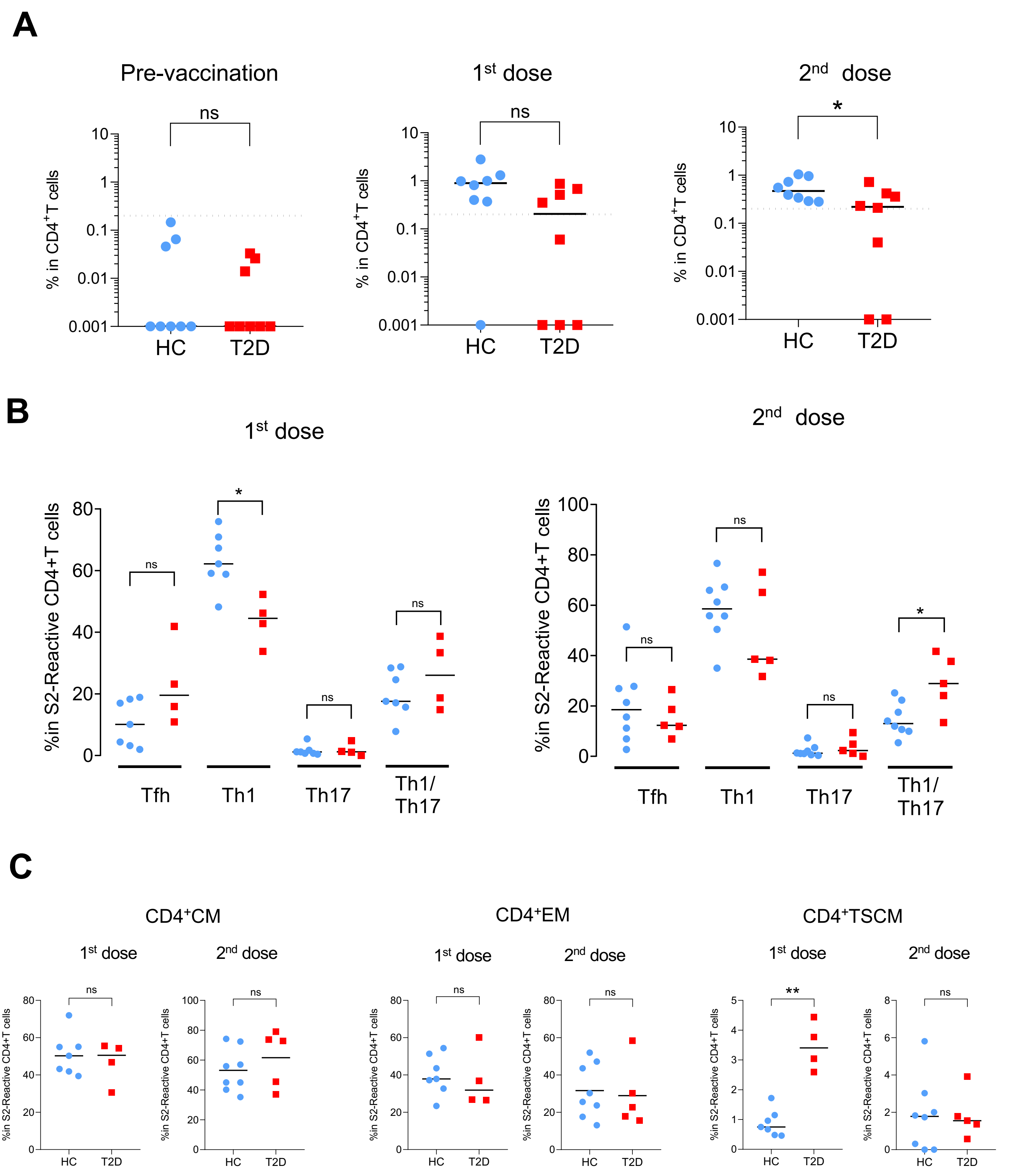

Supplementary Figure 9

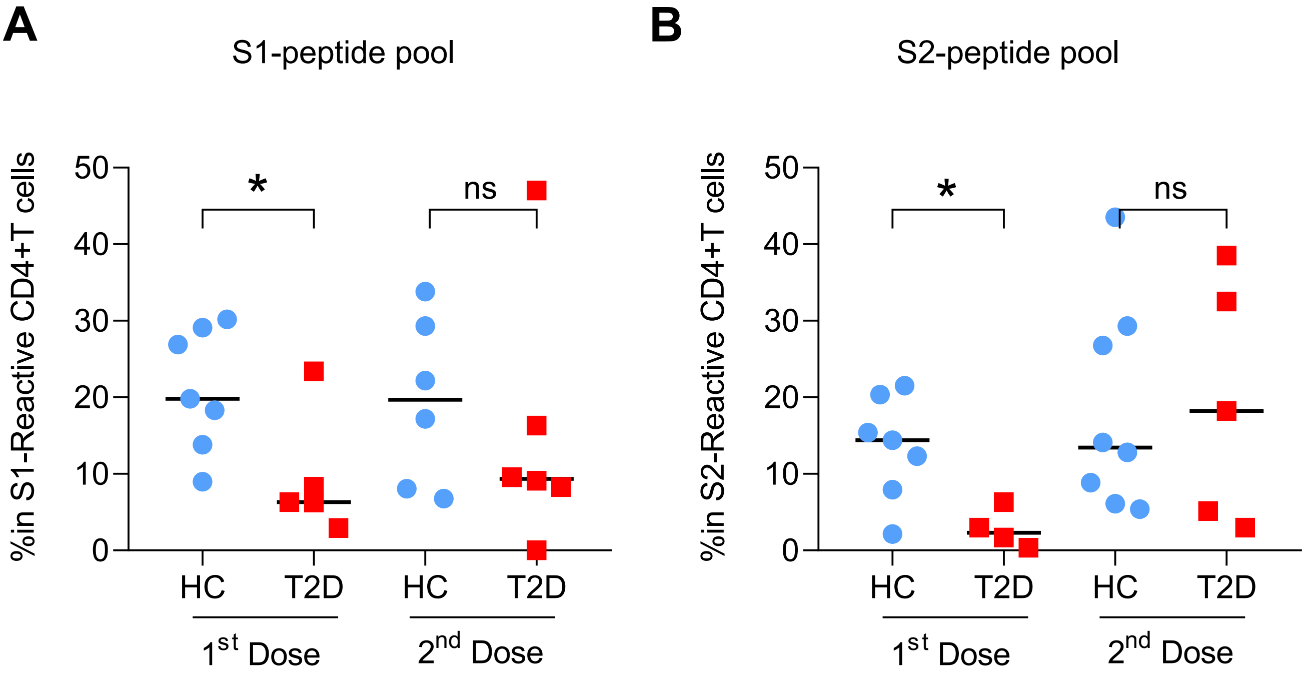

Supplementary Figure 10

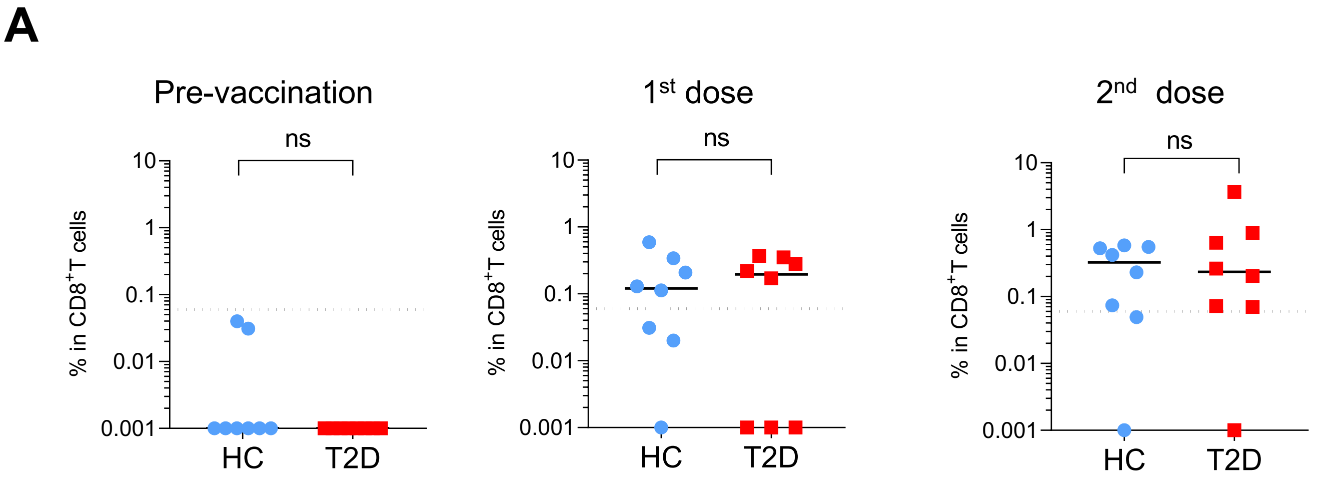

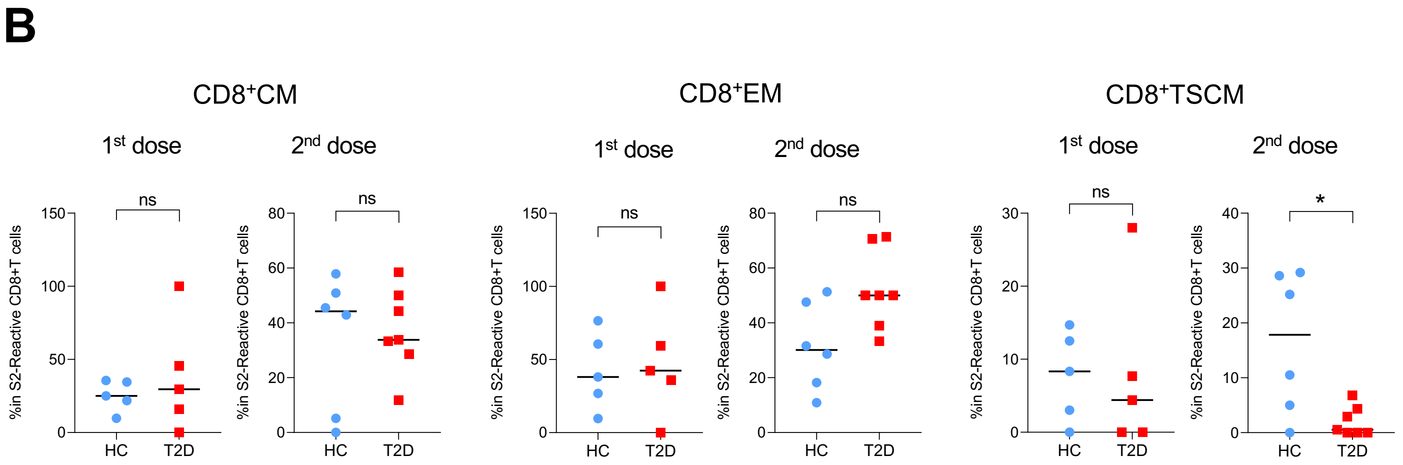

Supplementary Table 1

List of reagents

Reagent	Provider	Catalogue number	Dilution
**Antibodies**			
CD3 BV785	Biolegend	344842	1:100
CD4 APC-Cy7	Biolegend	344616	1:100
CD4 BV711	Biolegend	344648	1:200
CD8 AF700	Biolegend	344724	1:100
CD27 BV650	Biolegend	302827	1:40
CD45RA PE	Invitrogen	12-0458-42	1:200
CD95 AF488	Biolegend	305616	1:40
CD200 BV605	Biolegend	329218	1:20
CD40L APC/Fire™ 750	Biolegend	310848	1:20
CCR6 APC	Biolegend	353415	1:40
CCR7 Pacific Blue	Biolegend	353210	1:20
CXCR3 BV421	Biolegend	353715	1:40
CXCR5 PE	Invitrogen	12-9185-41	1:40
PD1 FITC	Biolegend	367411	1:40
CD137 AF647	Biolegend	309824	1:40
TNFα APC	Biolegend	502912	1:100
IFNγ PE-Cy7	Biolegend	502528	1:40
GZMB PE	Biolegend	372208	1:100
CD107a AF488	Biolegend	328610	1:100
APC Mouse IgG1	Biolegend	400119	1:40
Brilliant Violet 650™ Mouse IgG1	Biolegend	400163	1:40
PE Mouse IgG2b	Biolegend	400311	1:40
Brilliant Violet 605™ Mouse IgG1	Biolegend	400161	1:20
Alexa Fluor® 647 Mouse IgG1	Biolegend	400130	1:40
Brilliant Violet 421™ Mouse IgG1	Biolegend	400157	1:40
APC/Fire™ 750 Mouse IgG1	Biolegend	400195	1:20
APC Mouse IgG2b	Biolegend	400319	1:40
Pacific Blue™ Mouse IgG2a	Biolegend	400235	1:20
Alexa Fluor® 488 Mouse IgG1	Biolegend	400132	1:40

Reagent	Provider	Catalogue number
**Peptides**		
PepMix™ SARS-CoV-2	JPT Peptide Technologies	PM-WCPV-S-1
**Other**		
Lymphoprep™	STEMCELL Technologies	07861
Zombie Aqua™ Fixable Viability Kit	Biolegend	423102
Human TruStain FcX	Biolegend	422302
BD CellFIX (10x concentrate)	BD Bioscience	340181
Fixation/Permeabilization Kit	BD Bioscience	554714
Monensin Solution	Biolegend	420701
Brefeldin A Solution	Biolegend	420601
Ultra-LEAF™ Purified anti-human CD28 Antibody	Biolegend	302934
SARS-CoV-2 S1RBD IgG ELISA Kit [CE-IVD]	ImmunoDiagnostics	41A235
SARS-CoV-2 Surrogate Virus Neutralization Test (sVNT) Kit	GenScript	L00847-A
